# Statins and Renin Angiotensin System Inhibitors Dose-Dependently Protect Hypertensive Patients against Dialysis Risk

**DOI:** 10.1371/journal.pone.0162588

**Published:** 2016-09-15

**Authors:** Ju-Chi Liu, Yi-Ping Hsu, Szu-Yuan Wu

**Affiliations:** 1 Division of Cardiovascular Medicine, Department of Internal Medicine, Shuang Ho Hospital, Taipei Medical University, New Taipei City, Taiwan; 2 Institute of Toxicology, College of Medicine, National Taiwan University, Taipei, Taiwan; 3 Department of Radiation Oncology, Wan Fang Hospital, Taipei Medical University, Taipei, Taiwan; 4 Department of Internal Medicine, School of Medicine, College of Medicine, Taipei Medical University, Taipei, Taiwan; 5 Department of Biotechnology, Hungkuang University, Taichung, Taiwan; Istituto Di Ricerche Farmacologiche Mario Negri, ITALY

## Abstract

**Background:**

Taiwan has the highest renal disease incidence and prevalence in the world. We evaluated the association of statin and renin–angiotensin system inhibitor (RASI) use with dialysis risk in hypertensive patients.

**Methods:**

Of 248,797 patients who received a hypertension diagnosis in Taiwan during 2001–2012, our cohort contained 110,829 hypertensive patients: 44,764 who used RASIs alone; 7,606 who used statins alone; 27,836 who used both RASIs and statins; and 33,716 who used neither RASIs or statins. We adjusted for the following factors to reduce selection bias by using propensity scores (PSs): age; sex; comorbidities; urbanization level; monthly income; and use of nonstatin lipid-lowering drugs, metformin, aspirin, antihypertensives, diuretics, and beta and calcium channel blockers. The statin and RASI use index dates were considered the hypertension confirmation dates. To examine the dose–response relationship, we categorized only statin or RASI use into four groups in each cohort: <28 (nonusers), 28–90, 91–365, and >365 cumulative defined daily doses (cDDDs).

**Results:**

In the main model, PS-adjusted hazard ratios (aHRs; 95% confidence intervals [CIs]) for dialysis risk were 0.57 (0.50–0.65), 0.72 (0.53–0.98), and 0.47 (0.41–0.54) in the only RASI, only statin, and RASI + statin users, respectively. RASIs dose-dependently reduced dialysis risk in most subgroups and in the main model. RASI use significantly reduced dialysis risk in most subgroups, regardless of comorbidities or other drug use (*P* < 0.001). Statins at >365 cDDDs protected hypertensive patients against dialysis risk in the main model (aHR = 0.62, 95% CI: 0.54–0.71), regardless of whether a high cDDD of RASIs, metformin, or aspirin was used.

**Conclusion:**

Statins and RASIs independently have a significant dose-dependent protective effect against dialysis risk in hypertensive patients. The combination of statins and RASIs can additively protect hypertensive patients against dialysis risk.

## Introduction

In Taiwan, 92.4% of patients with renal diseases undergo hemodialysis; this percentage is 91.7% in the United States and 18.7% in Hong Kong [1]. The mean total lifetime treatment cost for dialysis patients is NT$6,112,755 ± NT$317,559 [2]. Furthermore, Taiwan has the highest incidence and prevalence of renal diseases and dialysis use worldwide [3]. The cost–effect problem in the Taiwanese National Health Insurance (NHI) system for dialysis use has emerged as a public health burden. Therefore, introducing an optimal therapy to avoid dialysis use among susceptible patients may aid in reducing national expenditure in the NHI program.

Hypertension, a major cause of renal diseases [3], is frequently seen in patients with acute and chronic renal diseases, particularly glomerular and vascular disorders [4]. Hypertension may primarily be caused by fluid overload, as indicated by a suppressed renin–angiotensin–aldosterone system and enhanced atrial natriuretic peptide release [5]. Hypertension is presented by 80%–85% of patients with chronic kidney disease (CKD) [6]. In patients with CKD, hypertension likely occurs because of a combination of factors including sodium retention, increased renin–angiotensin system activity, and enhanced sympathetic nervous system activity[7]. Hypertension is also common in acute vascular diseases, such as vasculitis and scleroderma renal crisis. In these settings, blood pressure increases because of ischemia-induced renin–angiotensin system activation, rather than volume expansion [8]. Renin–angiotensin system inhibitors (RASIs), including angiotensin-converting enzyme inhibitors (ACEIs), angiotensin II receptor blockers (ARBs), and direct renin inhibitors, are commonly used in hypertension treatment. Furthermore, inhibiting angiotensin II formation with an ACEI is effective in patients with vasculitis or scleroderma renal crisis [9]. In patients with proteinuric CKD, an ACEI or ARB is recommended in the first-line hypertension therapy [10–13]. However, no clear evidence indicating that early RASI use reduces dialysis risk in hypertensive patients without CKD has been reported.

Indirect evidence has indicated the beneficial effects of statins on vessel stiffening and endothelial function in patients with CKD [14, 15]. After renal injury, dyslipidemia may accelerate and perpetuate the yearly decline in the glomerular filtration rate (GFR) [16–18]; however, this effect has been confirmed through post hoc analyses, which can be limited by unmeasured confounders closely correlated with dyslipidemia [18, 19]. If present, the aforementioned effect is extremely uncertain and may require many trials to obtain conclusive results [20]. Two meta-analyses of small-scale randomized trials have demonstrated that statin therapy significantly alleviates albuminuria [21, 22]. However, the patients included in these trials were not uniformly using RASIs. By contrast, two large-scale randomized trials have revealed that statins do not affect albumin excretion in patients receiving optimal RASI therapy to reduce CKD progression and achieve satisfactory blood pressure control [23, 24]. Thus, conflicting data concerning the effect of statins on renal disease progression have been reported [11, 25–27]. Most data derived from large-scale intervention studies, with hard clinical endpoints, have suggested that statins do not prevent renal function loss [28–30]. All trials evaluating the effects of statin therapy on renal disease progression have used subset analyses of trials designed to evaluate the efficacy of statin therapy in treating cardiovascular disease in patients with CKD [31, 32]. However, experimental evidence has indicated that reducing lipid levels by using a drug such as lovastatin reduces renal injury progression [33–35].

Currently, statins and RASIs are not recommended for renal protection alone in hypertensive patients without CKD [36, 37]. In this study, we clarified the potential protective effects of statins and RASIs against dialysis risk in hypertensive patients without CKD.

## Materials and Methods

In Taiwan, the NHI program, established in 1995, currently provides comprehensive health insurance coverage to 98% of the population of more than 23 million people. We used data from the National Health Insurance Research Database (NHIRD). Distributions of age, sex, and health care costs in the NHIRD and among NHI enrollees do not differ significantly. Data that can be used to identify patients or care providers, including the names of medical institutions and physicians, are encrypted before being sent to the National Health Research Institutes (NHRI) for inclusion in the NHIRD. The NHRI further encrypts the data before releasing the database to researchers. Theoretically, the NHIRD data alone are insufficient to identify any individual. All researchers using the NHIRD and its data subsets must sign a written agreement declaring that they have no intention of attempting to obtain information that could potentially violate the privacy of patients or care providers [38–40].

**Fig 1:**
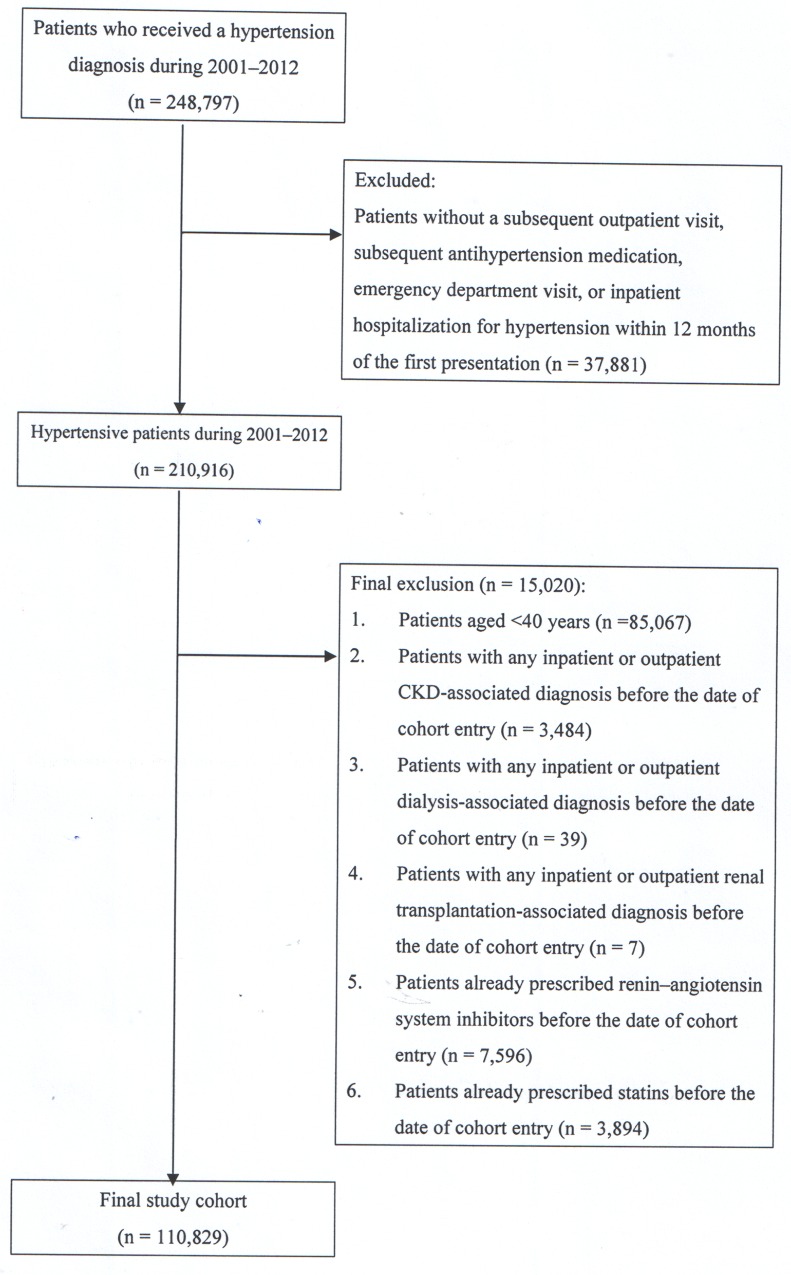
Data selection process.

Our study cohort comprised all patients who received a hypertension diagnosis (according to International Classification of Diseases, Ninth Revision, Clinical Modification [ICD-9-CM] codes) at all health care facilities in Taiwan (n = 248,791) between January 1, 2001, and December 31, 2012. We excluded patients without subsequent outpatient visits, subsequent antihypertension medications, and emergency department visits or inpatient hospitalizations for hypertension within 12 months of first presentation (n = 37,881), because these patients were considered to not have hypertension (Fig 1). In Taiwan, most dialysis patients are >40 years old [41] [2], and <40-year-old patients rarely receive a diagnosis of hypertension [42]. Thus, we focused only on patients aged >40 years. Consequently, we excluded 64,693 patients aged <40 years (n = 125,849), those with any inpatient or outpatient diagnosis associated with CKD before the date of cohort entry (n = 3,484), those with any inpatient or outpatient diagnosis associated with dialysis before the date of cohort entry (n = 39), those with any inpatient or outpatient diagnosis associated with renal transplantation before the date of cohort entry (n = 7), those having a RASI prescription before the date of cohort entry (n = 7,596), and those who had a statin prescription before the date of cohort entry (n = 3,894).

Our final study cohort comprised 110,829 patients with hypertension; of them, 44,764 used RASIs alone, 7,606 used statins alone, 27,836 used both RASIs and statins, and 33,716 used neither RASIs nor statins (Table 1). After literature review [43–47], we selected covariates on the basis of a logistic regression model. Each patient was followed to assess dialysis risk and protective factors. We evaluated the following demographic characteristics by using propensity scores (PSs): age; sex; diabetes; dyslipidemia; cerebrovascular disease; cardiovascular disease; hepatitis B and C virus infection; liver cirrhosis; moderate and severe liver disease; asthma; use of antihypertensives, diuretics, beta and calcium channel blockers, nonstatin lipid-lowering drugs, metformin, and aspirin; urbanization level; and monthly income (S1 Table). The index date of statin and RASI use was considered the confirmation date of hypertension. To evaluate the protective effects of statins and RASIs against dialysis risk in hypertensive patients, dialysis risk was considered the primary endpoint, whereas the secondary endpoints were the differential effects of various doses of and additive effect of RASIs and statins. The defined daily dose (DDD)—recommended by the World Health Organization—is a measure of the prescribed drug amount. The DDD is the assumed average maintenance dose per day of a drug consumed for its main indication in adults [40]. To examine the dose–response relationship, we categorized statin use into four groups in each cohort (<28, 28–90, 91–365, and >365 cumulative DDDs [cDDDs]) because the duration of the refill card was 3 months. Patients receiving <28 cDDDs were defined as nonusers (Tables 2–4) [48]. Furthermore, to examine the additive effect of RASI and statin use, we used sensitivity analysis of adjusted hazard ratios (aHRs) of RASIs and statins in reducing dialysis risk (Tables 2–4).

**Table 1:** Characteristics of the Sample Population.

	Nonuser(n = 30,633)		RASIs Alone(n = 44,764)		Statins Alone(n = 7,606)		RASIs + Statins(n = 27,826)		*P*
	n	%	n	%	N	%	n	%	
Age, years (mean ± SD)	58.82 (12.25)		58.77 (11.29)		57.69 (10.15)		57.49 (10.24)		<0.001
40–44	3727	12.17	5628	12.57	686	9.02	2920	10.49	<0.001
45–54	10237	33.42	14575	32.56	2800	36.81	9927	35.68	
55–64	7429	24.25	10718	23.94	2239	29.44	8128	29.21	
65–74	5460	17.82	8654	19.33	1419	18.66	5206	18.71	
≥75	3780	12.34	5189	11.59	462	6.07	1645	5.91	
Sex									
Female	14941	48.77	20154	45.02	4341	57.07	14106	50.69	<0.001
Male	15692	51.23	24610	54.98	3265	42.93	13720	49.31	
Comorbidities									
Diabetes	3868	12.63	6599	14.74	1477	19.42	6368	22.89	<0.001
Cerebrovascular disease	2645	8.63	3144	7.02	666	8.76	1833	6.59	<0.001
Dyslipidemia	5385	17.58	7151	15.97	2746	36.10	7507	26.98	<0.001
Cardiovascular disease	6869	22.42	8374	18.71	2060	27.08	5289	19.01	<0.001
Hepatitis B virus infection	1188	3.88	1917	4.28	290	3.81	943	3.39	<0.001
Hepatitis C virus infection	1293	4.22	2251	5.03	220	2.89	822	2.95	<0.001
Cirrhosis	1421	4.64	2183	4.88	203	2.67	771	2.77	<0.001
Moderate and severe liver disease	615	2.01	785	1.75	66	0.87	226	0.81	<0.001
Asthma	3526	11.51	4701	10.50	903	11.87	2626	9.44	<0.001
Antihypertension medications									
Antihypertensives	3851	12.57	8442	18.86	915	12.03	5710	20.52	<0.001
Diuretics	9347	30.51	28278	63.17	2324	30.55	19061	68.50	<0.001
Beta blockers	13834	45.16	25181	56.25	4488	59.01	18350	65.95	<0.001
Calcium channel blockers	19780	64.57	35057	78.32	5474	71.97	23055	82.85	<0.001
Comedication									
Nonstatin lipid-lowering drugs									
<28 cDDDs	29135	95.11	40833	91.22	5850	76.91	19970	71.77	<0.001
28–365 cDDDs	1215	3.97	2887	6.45	1278	16.80	5222	18.77	
>365 cDDDs	283	0.92	1044	2.33	478	6.28	2634	9.47	
Metformin									
<28 cDDDs	28230	92.16	36983	82.62	5805	76.32	16358	58.79	<0.001
28–365 cDDDs	1174	3.83	2774	6.20	610	8.02	2710	9.74	
>365 cDDDs	1229	4.01	5007	11.19	1191	15.66	8758	31.47	
Aspirin									
<28 cDDDs	24824	81.04	28498	63.66	4492	59.06	12112	43.53	<0.001
28–365 cDDDs	3706	12.10	8698	19.43	1589	20.89	6064	21.79	
>365 cDDDs	2103	6.87	7568	16.91	1525	20.05	9650	34.68	
Urbanization level									
Urban	22124	72.22	32232	72.00	5839	76.77	21229	76.29	<0.001
Suburban	5895	19.24	8603	19.22	1249	16.42	4728	16.99	
Rural	2614	8.53	3929	8.78	518	6.81	1869	6.72	
Monthly income (NT$)									
0	2028	6.62	2924	6.53	478	6.28	1798	6.46	<0.001
1–20,100	7008	22.88	10112	22.59	1544	20.30	5905	21.22	
20,100–30,300	11174	36.48	16147	36.07	2605	34.25	9760	35.08	
≥30,301	10423	34.03	15581	34.81	2979	39.17	10363	37.24	

cDDDs, cumulative defined daily doses; RASI, renin–angiotensin system inhibitor; SD, standard deviation.

**Table 2:** Sensitivity Analysis of aHRs of RASIs and Statins in the Reduction of Dialysis Risk.

	Nonusers (n = 29,806)	RASIs Alone (n = 44,857)	Statins Alone (n = 7,573)	RASIs + Statins (n = 28,593)
	aHR (95%CI)	aHR (95%CI)	aHR (95%CI)	aHR (95%CI)
**Main model†**	1.00	0.57(0.50–0.65)***	0.72(0.53–0.98)*	0.47(0.41–0.54)***
**Subgroup effects**				
Age, years				
<65	1.00	0.58(0.48–0.69)***	0.98(0.61–1.58)	0.47(0.40–0.57)***
≥65	1.00	0.58(0.47–0.71)***	0.68(0.44–1.03)	0.44(0.35–0.56)***
Sex				
Female	1.00	0.50(0.40–0.62)***	0.77(0.50–1.18)	0.43(0.35–0.54)***
Male	1.00	0.63(0.53–0.76)***	0.66(0.42–1.04)	0.50(0.42–0.60)***
Diabetes				
No	1.00	0.52(0.44–0.61)***	0.76(0.52–1.12)	0.37(0.31–0.45)***
Yes	1.00	0.61(0.46–0.80)***	0.60(0.35–1.03)	0.50(0.39–0.65)***
Cardiovascular disease				
No	1.00	0.58(0.50–0.68)***	0.77(0.54–1.09)	0.50(0.43–0.58)***
Yes	1.00	0.49(0.35–0.68)***	0.49(0.25–0.96)*	0.33(0.23–0.46)***
Cerebrovascular disease				
No	1.00	0.55(0.48–0.64)***	0.76(0.55–1.06)	0.45(0.39–0.52)***
Yes	1.00	0.71(0.46–1.08)	0.51(0.20–1.32)	0.60(0.39–0.94)*
Asthma				
No	1.00	0.57(0.50–0.66)***	0.71(0.52–0.98)*	0.47(0.40–0.54)***
Yes	1.00	0.55(0.36–0.86)**	2.83(0.38–21.41)	0.43(0.27–0.69)***
Antihypertensives				
No (<28 cDDDs)	1.00	0.57(0.49–0.66)***	0.75(0.53–1.07)	0.48(0.40–0.56)***
Yes (≥28 cDDDs)	1.00	0.64(0.47–0.87)**	0.70(0.37–1.34)	0.53(0.39–0.72)***
Diuretics				
No (<28 cDDDs)	1.00	0.55(0.44–0.70)***	0.67(0.40–1.13)	0.44(0.33–0.57)***
Yes (≥28 cDDDs)	1.00	0.61(0.52–0.73)***	0.78(0.53–1.16)	0.52(0.44–0.62)***
Beta blockers				
No (<28 cDDDs)	1.00	0.54(0.44–0.65)***	0.60(0.39–0.94)*	0.46(0.38–0.57)***
Yes (≥28 cDDDs)	1.00	0.63(0.52–0.77)***	0.91(0.58–1.41)	0.51(0.42–0.63)***
Calcium channel blockers				
No (<28 cDDDs)	1.00	0.68(0.53–0.86)**	0.57(0.32–1.01)	0.50(0.38–0.67)***
Yes (≥28 cDDDs)	1.00	0.57(0.48–0.67)***	0.82(0.56–1.19)	0.49(0.41–0.58)***
Nonstatin lipid-lowering drugs				
<28 cDDDs	1.00	0.56(0.49–0.65)***	0.78(0.56–1.10)	0.48(0.41–0.56)***
28–365 cDDDs	1.00	1.16(0.53–2.56)	1.30(0.45–3.74)	0.91(0.42–1.98)
>365 cDDDs	1.00	1.74(0.22–13.67)	1.93(0.17–21.42)	1.59(0.22–11.65)
Metformin				
<28 cDDDs	1.00	0.56(0.48–0.66)***	0.72(0.50–1.05)	0.46(0.38–0.56)***
28–365 cDDDs	1.00	0.78(0.53–1.15)	0.83(0.37–1.87)	0.69(0.48–0.99) *
>365 cDDDs	1.00	0.72(0.45–1.17)	1.05(0.46–2.39)	0.58(0.36–0.93)*
Aspirin				
<28 cDDDs	1.00	0.61(0.52–0.72)***	0.82(0.54–1.24)	0.53(0.44–0.63)***
28–365 cDDDs	1.00	0.50(0.36–0.70)***	0.68(0.36–1.29)	0.42(0.30–0.59)***
>365 cDDDs	1.00	0.75(0.46–1.23)	0.90(0.41–1.99)	0.64(0.39–1.04)

*: *P* < 0.05;

**: *P* < 0.01;

***: *P* < 0.001. CI, confidence interval; cDDDs, cumulative defined daily doses; aHR, adjusted hazard ratio; RASI, renin–angiotensin system inhibitor.

†Main model was propensity score adjusted for age; sex; diabetes; dyslipidemia; cerebrovascular disease; cardiovascular disease; hepatitis B and C virus infection; liver cirrhosis; moderate and severe liver disease; asthma; use of antihypertensives, diuretics, beta and calcium channel blockers, nonstatin lipid-lowering drugs, metformin, and aspirin; urbanization level; and monthly income.

**Table 3:** Sensitivity Analysis of the aHRs of RASIs in the Reduction of Dialysis Risk.

	RASI Nonusers	RASI Users			*P* for trend
		28–90 cDDDs	91–365 cDDDs	>365 cDDDs	
	aHR (95%CI)	aHR (95%CI)	aHR (95%CI)	aHR (95%CI)	
**Main model†**	1.00	0.78(0.65–0.92)**	0.73(0.63–0.85)***	0.47(0.41–0.54)***	<0.001
**Subgroup effects**					
Age, years					
< 65	1.00	0.78(0.61–0.98)*	0.68(0.56–0.83)***	0.47(0.39–0.56)***	<0.001
≥ 65	1.00	0.77(0.59–1.01)	0.78(0.62–0.99)*	0.47(0.38–0.58)***	<0.001
Sex					
Female	1.00	0.75(0.56–0.98)*	0.62(0.49–0.78)***	0.42(0.34–0.54)***	<0.001
Male	1.00	0.81(0.65–1.02)	0.83(0.69–1.02)	0.52(0.43–0.62)***	<0.001
Diabetes					
No	1.00	0.69(0.56–0.85)***	0.63(0.52–0.76)***	0.38(0.32–0.45)***	<0.001
Yes	1.00	1.10(0.80–1.52)	0.88(0.67–1.15)	0.60(0.47–0.76)***	<0.001
Cardiovascular disease					
No	1.00	0.80(0.66–0.97)*	0.75(0.64–0.89)***	0.48(0.42–0.56)***	<0.001
Yes	1.00	0.68(0.45–1.03)	0.67(0.47–0.95)*	0.41(0.30–0.57)***	<0.001
Cerebrovascular disease					
No	1.00	0.77(0.64–0.93)**	0.70(0.60–0.82)***	0.45(0.39–0.51)***	<0.001
Yes	1.00	0.81(0.46–1.41)	1.20(0.70–2.06)	0.67(0.45–0.99)*	0.056
Asthma					
No	1.00	0.83(0.69–1.00)*	0.74(0.63–0.86)***	0.48(0.41–0.55)***	<0.001
Yes	1.00	0.52(0.29–0.91)*	0.67(0.41–1.12)	0.43(0.27–0.67)***	<0.001
Antihypertensives					
No (<28 cDDDs)	1.00	0.84(0.69–1.02)	0.76(0.64–0.90)**	0.44(0.38–0.52)***	<0.001
Yes (≥28 cDDDs)	1.00	0.65(0.44–0.96)*	0.78(0.57–1.07)	0.61(0.46–0.80)***	<0.001
Diuretics					
No (<28 cDDDs)	1.00	0.92(0.69–1.22)	0.71(0.54–0.93)*	0.36(0.28–0.47)***	<0.001
Yes (≥28 cDDDs)	1.00	0.71(0.57–0.89)**	0.78(0.65–0.94)**	0.56(0.48, 0.66)***	<0.001
Beta blockers					
No (<28 cDDDs)	1.00	0.90(0.71–1.15)	0.68(0.55–0.84)***	0.43(0.35–0.52)***	<0.001
Yes (≥28 cDDDs)	1.00	0.69(0.53–0.89)**	0.80(0.65–0.99)*	0.54(0.45–0.65)***	<0.001
Calcium channel blockers					
No (<28 cDDDs)	1.00	0.93(0.69–1.25)	0.89(0.66–1.20)	0.47(0.36–0.61)***	<0.001
Yes (≥28 cDDDs)	1.00	0.72(0.58–0.90)**	0.73(0.61–0.88)***	0.50(0.42–0.59)***	<0.001
Statin drugs					
<28 cDDDs	1.00	0.80(0.66–0.96)*	0.73(0.61–0.86)***	0.45(0.38–0.52)***	<0.001
28–365 cDDDs	1.00	0.50(0.27–0.93)*	0.64(0.42–1.00)*	0.46(0.31–0.70)***	<0.001
>365 cDDDs	1.00	1.48(0.51–4.28)	1.92(0.78–4.75)	1.09(0.50–2.38)	0.492
Nonstatin lipid-lowering drugs					
<28 cDDDs	1.00	0.74(0.61–0.88)***	0.72(0.62–0.85)***	0.47(0.40–0.54)***	<0.001
28–365 cDDDs	1.00	1.66(0.78–3.49)	1.03(0.55–1.93)	0.73(0.41–1.31)	0.051
>365 cDDDs	1.00	11.33(1.68–76.24)*	4.66(1.12–19.35)*	1.54(0.45–5.23)	0.691
Metformin					
<28 cDDDs	1.00	0.76(0.62–0.92)**	0.74(0.61–0.88)**	0.42(0.35–0.49)***	<0.001
28–365 cDDDs	1.00	1.10(0.67–1.80)	0.90(0.62–1.31)	0.66(0.46–0.94)*	0.005
>365 cDDDs	1.00	0.83(0.48–1.45)	0.70(0.44–1.11)	0.61(0.41–0.91)*	0.007
Aspirin					
<28 cDDDs	1.00	0.88(0.72–1.08)	0.76(0.63–0.91)**	0.46(0.39–0.54)***	<0.001
28–365 cDDDs	1.00	0.54(0.36–0.81)**	0.63(0.45–0.86)**	0.45(0.33–0.61)***	<0.001
>365 cDDDs	1.00	0.71(0.37–1.37)	1.07(0.66–1.76)	0.70(0.47–1.06)	0.084

*: *P* < 0.05;

**: *P* < 0.01;

***: *P* < 0.001. CI, confidence interval; cDDD, cumulative defined daily dose; aHR, adjusted hazard ratio; RASI, renin–angiotensin system inhibitor.

†Main model was propensity score adjusted for age; sex; diabetes; dyslipidemia; cerebrovascular disease; cardiovascular disease; hepatitis B and C virus infection; liver cirrhosis; moderate and severe liver disease; asthma; use of antihypertensives, diuretics, beta and calcium channel blockers, nonstatin lipid-lowering drugs, metformin, aspirin, and statins; urbanization level; and monthly income.

**Table 4:** Sensitivity Analysis of the aHRs of Statins in the Reduction of Dialysis Risk.

	Statin Non-User	Statin User			*P* for Trend
		28–90 cDDDs	91–365 cDDDs	>365 cDDDs	
	aHR (95%CI)	aHR (95%CI)	aHR (95%CI)	aHR (95%CI)	
**Main model†**	1.00	1.02(0.85–1.23)	1.00(0.86, 1.16)	0.62(0.54–0.71)***	<0.001
**Subgroup effects**					
Age, years					
<65	1.00	1.06(0.82–1.36)	0.97(0.80–1.16)	0.59(0.50–0.70)***	<0.001
≥65	1.00	0.98(0.74–1.30)	1.02(0.80–1.31)	0.59(0.46–0.76)***	<0.001
Sex					
Female	1.00	0.98(0.75–1.30)	1.06(0.85–1.33)	0.61(0.50–0.74)***	<0.001
Male	1.00	1.05(0.82–1.36)	0.95(0.78–1.17)	0.63(0.52–0.76)***	<0.001
Diabetes					
No	1.00	0.98(0.76–1.27)	0.93(0.75–1.15)	0.56(0.45–0.70)***	<0.001
Yes	1.00	1.08(0.82–1.43)	1.03(0.83–1.27)	0.59(0.49–0.70)***	<0.001
Cardiovascular disease					
No	1.00	1.04(0.85–1.27)	1.04(0.88–1.22)	0.64(0.56–0.75)***	<0.001
Yes	1.00	0.98(0.58–1.67)	0.83(0.57–1.19)	0.50(0.36–0.70)***	<0.001
Cerebrovascular disease					
No	1.00	1.01(0.83–1.23)	1.00(0.86–1.17)	0.62(0.54–0.71)***	<0.001
Yes	1.00	1.09(0.57–2.09)	1.06(0.64–1.76)	0.59(0.37–0.95)*	0.041
Asthma					
No	1.00	1.02(0.84–1.24)	0.98(0.84–1.15)	0.62(0.54–0.71)***	<0.001
Yes	1.00	0.93(0.45–1.91)	1.26(0.69–2.30)	0.56(0.32–0.96)*	0.072
Antihypertensives					
No (<28 cDDDs)	1.00	1.05(0.83–1.33)	1.06(0.88–1.27)	0.64(0.54–0.75)***	<0.001
Yes (≥28 cDDDs)	1.00	1.03(0.75–1.40)	0.93(0.71–1.21)	0.60(0.47–0.76)***	<0.001
Diuretics					
No (<28 cDDDs)	1.00	0.95(0.62–1.45)	0.93(0.68–1.25)	0.53(0.39–0.71)***	<0.001
Yes (≥28 cDDDs)	1.00	1.05(0.85–1.29)	1.04(0.88–1.24)	0.66(0.56–0.77)***	<0.001
Beta blockers					
No (<28 cDDDs)	1.00	1.03(0.76–1.40)	1.06(0.84–1.33)	0.61(0.48–0.77)***	<0.001
Yes (≥28 cDDDs)	1.00	1.01(0.80–1.28)	0.97(0.80–1.17)	0.63(0.53–0.75)***	<0.001
Calcium channel blockers					
No (<28 cDDDs)	1.00	0.90(0.61–1.34)	0.97(0.65–1.45)	0.50(0.35–0.71)***	<0.001
Yes (≥28 cDDDs)	1.00	1.04(0.84–1.29)	1.03(0.88–1.21)	0.65(0.56–0.76)***	<0.001
RASIs					
<28 cDDDs	1.00	1.17(0.79–1.72)	1.09(0.75–1.57)	0.56(0.39–0.80)**	0.009
28–365 cDDDs	1.00	0.89(0.67–1.19)	0.90(0.72–1.14)	0.74(0.57–0.97)*	0.032
>365 cDDDs	1.00	1.11(0.80–1.54)	1.14(0.89–1.45)	0.66(0.54–0.80)***	<0.001
Nonstatin lipid-lowering drugs					
<28 cDDDs	1.00	1.06(0.86–1.29)	0.99(0.84–1.17)	0.64(0.55–0.76)***	<0.001
28–365 cDDDs	1.00	0.68(0.38–1.21)	1.07(0.70–1.62)	0.71(0.51–0.99)*	0.079
>365 cDDDs	1.00	2.29(0.49–10.72)	1.88(0.88–4.02)	0.93(0.46–1.88)	0.706
Metformin					
<28 cDDDs	1.00	1.08(0.84–1.40)	0.94(0.76–1.17)	0.59(0.46–0.75)***	<0.001
28–365 cDDDs	1.00	0.83(0.56–1.21)	1.19(0.85–1.67)	0.82(0.60–1.12)	0.434
>365 cDDDs	1.00	0.98(0.66–1.46)	1.07(0.81–1.42)	0.59(0.47–0.73)***	<0.001
Aspirin					
<28 cDDDs	1.00	1.08(0.83–1.41)	1.03(0.83–1.27)	0.68(0.55–0.84)***	0.003
28–365 cDDDs	1.00	0.82(0.59–1.12)	1.01(0.77–1.32)	0.64(0.49–0.84)**	0.005
>365 cDDDs	1.00	1.35(0.83–2.20)	1.06(0.76–1.49)	0.65(0.50–0.85)**	0.001

*: *P* < 0.05;

**: *P* < 0.01;

***: *P* < 0.001. CI, confidence interval; cDDD, cumulative defined daily dose; aHR, adjusted hazard ratio; RASI, renin–angiotensin system inhibitor.

†Main model was propensity score adjusted for age; sex; diabetes; dyslipidemia; cerebrovascular disease; cardiovascular disease; hepatitis B and C virus infection; liver cirrhosis; moderate and severe liver disease; asthma; use of antihypertensives, diuretics, beta and calcium channel blockers, nonstatin lipid-lowering drugs, metformin, aspirin, and RASIs; urbanization level; and monthly income.

PSs were derived using a logistic regression model to estimate the effect of RASIs and statins by accounting for the covariates predicting intervention (statins and RASIs) receipt. All potential confounders were included in the list of regressors (C statistic: 0.684). This method is used in observational studies to reduce selection bias [49]. The following covariates in the main model were adjusted according to the PS: age; sex; diabetes; dyslipidemia; cerebrovascular disease; cardiovascular disease; hepatitis B and C virus infection; liver cirrhosis; moderate and severe liver disease; asthma; use of antihypertensives, diuretics, beta and calcium channel blockers, nonstatin lipid-lowering drugs, metformin, and aspirin; urbanization level; and monthly income (NT$0, NT$1–NT$21,000, NT$21,000–NT$33,300; and ≥NT$33,301; Table 2). The endpoint for users of RASIs alone, statins alone, and RASIs + statins and nonusers was the recommendation of dialysis (ICD-9-CM V45.11 or V45.12), with a subsequent outpatient visit, emergency department visit, or inpatient hospitalization for any dialysis treatment within 12 months of diagnosis; nonusers were treated as the reference arm.

A time-dependent Cox proportional hazard model was used to calculate the HRs of dialysis risk in the users of RASIs alone, statins alone, and RASI + statin and nonusers. In the multivariate analysis, the HRs were adjusted for the aforementioned covariates. All analyses were conducted using SAS version 9.3 (SAS, Cary, NC, USA); two-tailed test results with *P* < 0.05 were considered significant. In sensitivity analyses, external adjustments are used to improve the understanding of the effects of drugs and other covariates in epidemiological database studies [50]. Hence, in our sensitivity analysis, data were adjusted in different models to estimate the association of dialysis incidence with age; sex; diabetes; dyslipidemia; cerebrovascular disease; cardiovascular disease; hepatitis B and C virus infection; liver cirrhosis; moderate and severe liver disease; asthma; and use of antihypertensives, diuretics, beta and calcium channel blockers, nonstatin lipid-lowering drugs, statins, RASIs, metformin, and aspirin.

## Results and Discussion

Compared with the users of other drugs, only statin users exhibited a higher prevalence of preexisting medical comorbidities including cerebrovascular disease, cardiovascular disease, and dyslipidemia (all *P* < 0.001). In addition, significant differences were observed among the four groups in the distributions of age; sex; monthly income; urbanization level; and use of nonstatin lipid-lowering drugs, aspirin, RASIs, and metformin (Table 1). A higher proportion of nonusers used nonstatin lipid-lowering drugs, metformin, and aspirin at <28 cDDDs; however, most RASI or statin users used these drugs at >365 cDDDs. A lower proportion of statin nonusers had a monthly income of ≥NT$33,301 or resided in urban areas.

In the sensitivity analysis, PS adjustments were made to estimate the associations of age; sex; diabetes; dyslipidemia; cerebrovascular disease; cardiovascular disease; hepatitis B and C virus infection; liver cirrhosis; moderate and severe liver disease; asthma; and use of antihypertensives, diuretics, beta and calcium channel blockers, nonstatin lipid-lowering drugs, metformin, and aspirin with the incidence of dialysis in different models. Table 2 shows that the effects of the use of RASIs alone, statins alone, or RASIs + statins remained significant in the different groups when the main model was PS adjusted. A stratified sensitivity analysis was performed to evaluate the dialysis risk among the users of different drugs. After PS adjustments for the main model, aHRs (95% confidence intervals [CIs]) of dialysis were 0.57 (0.50–0.65) for those using RASIs alone, 0.72 (0.53–0.98) for those using statins alone, and 0.47 (0.41–0.54) for those using RASIs + statins (Table 2). Table 2 also shows that the effects of RASI + statin use remained significant in the subgroups of various covariates namely age; sex; diabetes; cerebrovascular disease; cardiovascular disease; asthma; and use of antihypertensives, diuretics, beta and calcium channel blockers, and metformin. The combined use of RASIs and statins might have the highest potential for reducing dialysis risk, as indicated by the RASI + statin group having the lowest aHR among all groups. The effects were nonsignificant in users of RASIs alone, statins alone, and RASIs + statins when the cDDDs of nonstatin lipid-lowering drugs were moderate to high (>28) or when those of aspirin were high (>365). When the dose of metformin was moderate to high, the effects were significant only for the RASI + statin group.

RASIs dose-dependently reduced dialysis risk in most subgroups and the main model (Table 3). All aHRs indicated that RASI use caused significant reductions in dialysis risk in most subgroups, regardless of comorbidities or drug use (*P* < 0.001). Our data revealed that RASI use, with a dose-dependent effect frequency, has a protective effect against dialysis risk, which was particularly predominant in female patients using RASIs at >365 cDDDs (aHR = 0.42, 95% CI: 0.34–0.54) and in patients without diabetes who used RASIs at >365 cDDDs (aHR = 0.38, 95% CI: 0.32–0.45), those without cerebrovascular disease who used RASIs at >365 cDDDs (aHR = 0.45, 95% CI: 0.39–0.51), and those not using antihypertensives, diuretics, beta and calcium channel blockers, statins, nonstatin lipid-lowering drugs, metformin, or aspirin (*P* < 0.001). If the patients used high cDDDs of statins, nonstatin lipid-lowering drugs, or aspirin, no protective effect against dialysis risk was observed, even for high RASI cDDDs. If the patients used high cDDDs of nonstatin lipid-lowering drugs, no protective effect against dialysis risk was noted, even when RASIs were used; in addition, the aHRs significantly increased with the dose of nonstatin lipid-lowering drugs, but not with the dose of RASIs. As presented in Table 4, we performed a sensitivity analysis with PS adjustments in the main model for age; sex; diabetes; dyslipidemia; cerebrovascular disease; cardiovascular disease; hepatitis B and C virus infection; liver cirrhosis; moderate and severe liver disease; asthma; urbanization level; monthly income; and use of antihypertensives, diuretics, beta and calcium channel blockers, RASIs, nonstatin lipid-lowering drugs, metformin, and aspirin. Statins at >365 cDDDs conferred a protective effect against dialysis risk in hypertensive patients in the main model (aHR = 0.62, 95% CI: 0.54–0.71). This protective effect was more predominant in patients with cerebrovascular disease (aHR = 0.59, 95% CI: 0.37, 0.59), those with asthma (aHR = 0.56, 95% CI: 0.32, 0.96), and those not using diuretics, beta and calcium channel blockers, RASIs, nonstatin lipid-lowering drugs, metformin, or aspirin (all *P* < 0.001). If patients used high cDDDs of nonstatin lipid-lowering drugs, no protective effect against dialysis risk was observed, even for high cDDD statin use; furthermore, aHRs increased nonsignificantly with the dose of nonstatin lipid-lowering drugs. Therefore, regardless of whether high cDDDs of RASIs, metformin, or aspirin were used, high cDDDs of statins had a protective effect against dialysis risk in the statin alone group.

RASIs are used in first-line antihypertensive therapy in all patients with heart failure or asymptomatic left ventricle dysfunction, those with anterior myocardial infarction and diabetes or systolic dysfunction, and those with proteinuric CKD [51, 52]. RASIs have a cardioprotective effect, independent of blood pressure lowering noted in patients at a high risk of cardiovascular events. Hypertension can be a causative or contributory factor in kidney disease development [53]. No data are available for estimating the protective effect of RASIs in reducing dialysis risk in hypertensive patients without CKD. According to our research, our study is the first to report a dose-dependent effect of RASIs in reducing dialysis risk in hypertensive patients without CKD. We observed that in addition to their cardioprotective effect, RASIs have a protective effect against dialysis risk in hypertensive patients. The use of RASIs dose-dependently reflected their protective effect against dialysis risk in hypertensive patients without CKD (Table 3).

Hyperlipidemia is common in patients with renal diseases, particularly nephrotic syndrome [54]. In addition to accelerating systemic atherosclerosis development, experimental studies have suggested that high lipid levels also may promote renal disease progression [55, 56]. The major experimental evidence supporting this hypothesis in animals is that loading cholesterol increases glomerular injury and that reducing lipid levels by using statins slows injury progression [33–35]. Furthermore, the beneficial lipid-lowering effect may be supplementary to that of blood pressure lowering, as observed in some renal disease models [34]. However, the factors responsible for the lipid-lowering effects remain unclear. In various animal models, high cholesterol intake can be deleterious, causing an increase in intraglomerular pressure [35]; by contrast, lipid-lowering drugs, which do not affect glomerular hemodynamics, can be more beneficial [56]. These contradictory observations suggest that in addition to intraglomerular pressure, other mechanisms may contribute to blood pressure lowering. Moreover, statins may act independent of plasma lipid levels by directly inhibiting mesangial cell proliferation and monocyte chemoattractant production [57, 58]. The applicability of these findings to human diseases is uncertain; hence, our present human data are valuable. Numerous secondary analyses of data from lipid-related trials have suggested that high lipid levels accelerate renal disease progression, whereas statins delay this progression. In the current study, statins at >365 cDDDs conferred an independent protective effect against dialysis risk to hypertensive patients (aHR = 0.62, 95% CI: 0.54–0.71) in the PS-adjusted main model (Table 4). The combined use of RASIs and statins may have the highest potential in reducing dialysis risk with the smallest aHR (0.47, 95% CI: 0.41–0.54) compared with that of the use of RASIs alone or statins alone (Table 2). The current data regarding the additive effect of dialysis risk reduction in humans corroborate those of a preclinical study [34]. The novelty of our study is the establishment of clinical data demonstrating that statins confer an independent protective effect against dialysis risk to hypertensive patients, and this can be further enhanced through combined use of statins and RASIs.

The effects of RASIs alone, statins alone, or RASIs + statins were nonsignificant when the cDDDs of nonstatin lipid-lowering drugs were moderate to high (>28) or when those of aspirin were high (>365). If a moderate-to-high dose of metformin was used, the effect was significant only when RASIs and statins were used in combination. A history of diabetes, hypertension, cerebrovascular disease, or cardiovascular disease can confer the highest CKD risk to patients [59]. In this study, the protective effect was more predominant in female patients, those without diabetes, and those without cerebrovascular disease (Table 2). These outcomes correspond to those of a previous study [59]. Our findings imply that early RASI and statin use by hypertensive patients without diabetes or cerebrovascular disease might strengthen its protective effect against dialysis risk.

Higher cDDDs of aspirin for cerebrovascular disease, metformin for diabetes, and nonstatin lipid-lowering drugs for hyperlipidemia might correspond to disease severity and duration, both of which cannot be PS adjusted. Thus, the severity and duration of comorbidities might mask the protective effect of RASIs or statins (Tables 2–4). The following was most predominantly observed in nonstatin lipid-lowering drug users: no protective effect against dialysis risk, even with RASI or statin use, and aHRs increased with an increase in the cDDDs of nonstatin lipid-lowering drug use (Tables 2–4); this is because poor hyperlipidemia control is a major risk factor for renal disease progression, according to preclinical studies [55, 56]. In addition, the protective effect against the dialysis risk of statins might be superior to that of nonstatin lipid-lowering drugs, according to the highest aHR (2.29) being observed in hypertensive patients using >365 cDDDs of nonstatin lipid-lowering drugs and 28–90 cDDDs of statins (Table 4). This is the first study with human clinical data demonstrating that statins have a stronger protective effect against dialysis risk than nonstatin lipid-lowering drugs do in hypertensive patients without CKD, potentially because statins directly inhibit mesangial cell proliferation and monocyte chemoattractant production [57, 58].

Regardless of whether high doses of RASIs, metformin, or aspirin are used, the protective effect against dialysis risk was observed with the use of high cDDDs of statins in the statin alone group (Table 4). These findings suggest that the pharmacological mechanism underlying the protective effect of statins is independent from that of RASIs. Although diabetes, hypertension, and cardiovascular disease are risk factors for CKD [59], statins have a significant protective effect, even when >365 cDDDs of metformin, aspirin, or RASIs are used (Table 4). By contrast, no protective effect is observed when >365 cDDDs of RASIs are used with >365 cDDDs of statins or aspirin (Table 3). Therefore, statins have independent protective and additive effects along with RASIs against dialysis risk in hypertensive patients. Two randomized trials have demonstrated that statins combined with angiotensin blockers do not slow CKD progression but lead to favorable blood pressure control [23, 24]. This may be because the doses and duration of statin use in these trials were insufficient. In the future, randomized trials considering higher doses and longer durations of statin use should be conducted.

This study has six limitations. First, different statin and RASI types were considered but not analyzed separately; thus, the potential effects of a specific statin or RASI remain unknown. Second, evidence from observational studies has suggested that lifestyle factors, particularly social, mental, and physical activities, are inversely associated with dialysis risk. However, methodological concerns may obscure the precise relationship between these factors and dialysis risk. In our study, we used PSs to match age, sex, comorbidities, urbanization level, and monthly income. Third, urbanization level and monthly income were used as unvalidated alternatives to lifestyle factors. To obtain such information regarding the actual factors, a large-scale randomized trial should be conducted along with a suitable regimen and appropriately selected patients to compare standard approaches. Fourth, in the present study, dialysis recommendation and comorbidity diagnoses were completely dependent on the ICD codes. Nevertheless, the NHI Administration randomly reviews medical records and interviews patients to validate diagnoses. Hospitals with outlier diagnoses and practices may be audited and subsequently heavily penalized if malpractice or discrepancies are discovered. Fifth, the NHIRD contains no information on several unmeasured confounders including body mass index, laboratory data, compliance with drug use, smoking status, alcohol intake, and use of other dialysis-associated over-the-counter drugs. However, if patient compliance is poor, the drug effects are underestimated, causing bias toward the null hypothesis [60]. Thus, in cases of poor patient compliance, the true effects of statins or RASIs may have been underestimated. Considering the magnitude and significance of the observed effects, it is unlikely that this limitation compromised the results. Finally, our study was not prospective, randomized, or blinded; hence, a cause–effect relationship could not be established. The findings of this study suggest that statins or RASIs independently exert a significant protective effect against dialysis risk in hypertensive patients in a dose-dependent manner. The combined use of statins and RASIs has an additive effect against dialysis risk in hypertensive patients. Additional randomized studies are warranted to verify our findings.

## Conclusions

Statins and RASIs independently exert a significant dose-dependent protective effect against dialysis risk in hypertensive patients without CKD. Statins in combination with RASIs can additively protect hypertensive patients against dialysis risk.

## Supporting Information

S1 Table Candidate Variables for the Logistic Regression Model. (DOCX)

## References

[1] Chang YK, Hsu CC, Hwang SJ, Chen PC, Huang CC, Li TC, et al A comparative assessment of survival between propensity score-matched patients with peritoneal dialysis and hemodialysis in Taiwan. Medicine (Baltimore). 2012;91(3):144–51. Epub 2012/04/25. 10.1097/MD.0b013e318256538e . 22525667

[2] Kao TW, Chang YY, Chen PC, Hsu CC, Chang YK, Chang YH, et al Lifetime costs for peritoneal dialysis and hemodialysis in patients in Taiwan. Peritoneal dialysis international: journal of the International Society for Peritoneal Dialysis. 2013;33(6):671–8. Epub 2013/05/03. 10.3747/pdi.2012.00081 23636434 PMC3862097

[3] Hwang SJ, Tsai JC, Chen HC. Epidemiology, impact and preventive care of chronic kidney disease in Taiwan. Nephrology (Carlton). 2010;15 Suppl 2:3–9. Epub 2010/07/09. 10.1111/j.1440-1797.2010.01304.x . 20586940

[4] Bakris GL, Ritz E. The message for World Kidney Day 2009: hypertension and kidney disease: a marriage that should be prevented. Kidney international. 2009;75(5):449–52. Epub 2009/02/17. 10.1038/ki.2008.694 . 19218998

[5] Rodriguez-Iturbe B, Colic D, Parra G, Gutkowska J. Atrial natriuretic factor in the acute nephritic and nephrotic syndromes. Kidney international. 1990;38(3):512–7. Epub 1990/09/01. . 2146429 10.1038/ki.1990.233

[6] Whaley-Connell AT, Sowers JR, Stevens LA, McFarlane SI, Shlipak MG, Norris KC, et al CKD in the United States: Kidney Early Evaluation Program (KEEP) and National Health and Nutrition Examination Survey (NHANES) 1999–2004. American journal of kidney diseases: the official journal of the National Kidney Foundation. 2008;51(4 Suppl 2):S13–20. Epub 2008/04/11. 10.1053/j.ajkd.2007.12.016 . 18359403

[7] Neumann J, Ligtenberg G, Klein II, Koomans HA, Blankestijn PJ. Sympathetic hyperactivity in chronic kidney disease: pathogenesis, clinical relevance, and treatment. Kidney international. 2004;65(5):1568–76. Epub 2004/04/17. 10.1111/j.1523-1755.2004.00552.x . 15086894

[8] Parra G, Rodriguez-Iturbe B, Colina-Chourio J, Garcia R. Short-term treatment with captopril in hypertension due to acute glomerulonephritis. Clinical nephrology. 1988;29(2):58–62. Epub 1988/02/01. . 3282729

[9] Catapano F, Chiodini P, De Nicola L, Minutolo R, Zamboli P, Gallo C, et al Antiproteinuric response to dual blockade of the renin-angiotensin system in primary glomerulonephritis: meta-analysis and metaregression. American journal of kidney diseases: the official journal of the National Kidney Foundation. 2008;52(3):475–85. Epub 2008/05/13. 10.1053/j.ajkd.2008.03.008 . 18468748

[10] Kent DM, Jafar TH, Hayward RA, Tighiouart H, Landa M, de Jong P, et al Progression risk, urinary protein excretion, and treatment effects of angiotensin-converting enzyme inhibitors in nondiabetic kidney disease. Journal of the American Society of Nephrology: JASN. 2007;18(6):1959–65. Epub 2007/05/04. 10.1681/ASN.2006101081 . 17475813

[11] Fink HA, Ishani A, Taylor BC, Greer NL, MacDonald R, Rossini D, et al Screening for, monitoring, and treatment of chronic kidney disease stages 1 to 3: a systematic review for the U.S. Preventive Services Task Force and for an American College of Physicians Clinical Practice Guideline. Annals of internal medicine. 2012;156(8):570–81. Epub 2012/04/18. 10.7326/0003-4819-156-8-201204170-00004 . 22508734

[12] Jafar TH, Schmid CH, Landa M, Giatras I, Toto R, Remuzzi G, et al Angiotensin-converting enzyme inhibitors and progression of nondiabetic renal disease. A meta-analysis of patient-level data. Annals of internal medicine. 2001;135(2):73–87. Epub 2001/07/17. . 11453706 10.7326/0003-4819-135-2-200107170-00007

[13] Casas JP, Chua W, Loukogeorgakis S, Vallance P, Smeeth L, Hingorani AD, et al Effect of inhibitors of the renin-angiotensin system and other antihypertensive drugs on renal outcomes: systematic review and meta-analysis. Lancet. 2005;366(9502):2026–33. Epub 2005/12/13. 10.1016/S0140-6736(05)67814-2 . 16338452

[14] Konstadinidou I, Boletis JN, Perrea D, Triantafyllou A, Filiopoulos V, Stamataki E, et al Beneficial effects of fluvastatin on progressive renal allograft dysfunction. Transplantation proceedings. 2003;35(4):1364–7. Epub 2003/06/27. . 12826160 10.1016/s0041-1345(03)00376-2

[15] Blacher J, Safar ME, Pannier B, Guerin AP, Marchais SJ, London GM. Prognostic significance of arterial stiffness measurements in end-stage renal disease patients. Current opinion in nephrology and hypertension. 2002;11(6):629–34. Epub 2002/10/24. 10.1097/01.mnh.0000040049.33359.2b . 12394609

[16] Schaeffner ES, Kurth T, Curhan GC, Glynn RJ, Rexrode KM, Baigent C, et al Cholesterol and the risk of renal dysfunction in apparently healthy men. Journal of the American Society of Nephrology: JASN. 2003;14(8):2084–91. Epub 2003/07/23. . 12874462 10.1681/ASN.V1482084

[17] Manttari M, Tiula E, Alikoski T, Manninen V. Effects of hypertension and dyslipidemia on the decline in renal function. Hypertension. 1995;26(4):670–5. Epub 1995/10/01. . 7558229 10.1161/01.hyp.26.4.670

[18] Rahman M, Yang W, Akkina S, Alper A, Anderson AH, Appel LJ, et al Relation of serum lipids and lipoproteins with progression of CKD: The CRIC study. Clinical journal of the American Society of Nephrology: CJASN. 2014;9(7):1190–8. Epub 2014/05/17. 10.2215/CJN.09320913 24832097 PMC4078958

[19] Chawla V, Greene T, Beck GJ, Kusek JW, Collins AJ, Sarnak MJ, et al Hyperlipidemia and long-term outcomes in nondiabetic chronic kidney disease. Clinical journal of the American Society of Nephrology: CJASN. 2010;5(9):1582–7. Epub 2010/06/19. 10.2215/CJN.01450210 20558558 PMC2974397

[20] Muntner P, Coresh J, Smith JC, Eckfeldt J, Klag MJ. Plasma lipids and risk of developing renal dysfunction: the atherosclerosis risk in communities study. Kidney international. 2000;58(1):293–301. Epub 2000/07/08. 10.1046/j.1523-1755.2000.00165.x . 10886574

[21] Douglas K, O'Malley PG, Jackson JL. Meta-analysis: the effect of statins on albuminuria. Annals of internal medicine. 2006;145(2):117–24. Epub 2006/07/19. . 16847294 10.7326/0003-4819-145-2-200607180-00009

[22] Strippoli GF, Navaneethan SD, Johnson DW, Perkovic V, Pellegrini F, Nicolucci A, et al Effects of statins in patients with chronic kidney disease: meta-analysis and meta-regression of randomised controlled trials. Bmj. 2008;336(7645):645–51. Epub 2008/02/27. 10.1136/bmj.39472.580984.AE 18299289 PMC2270960

[23] Atthobari J, Brantsma AH, Gansevoort RT, Visser ST, Asselbergs FW, van Gilst WH, et al The effect of statins on urinary albumin excretion and glomerular filtration rate: results from both a randomized clinical trial and an observational cohort study. Nephrology, dialysis, transplantation: official publication of the European Dialysis and Transplant Association—European Renal Association. 2006;21(11):3106–14. Epub 2006/05/25. 10.1093/ndt/gfl244 . 16720593

[24] Ruggenenti P, Perna A, Tonelli M, Loriga G, Motterlini N, Rubis N, et al Effects of add-on fluvastatin therapy in patients with chronic proteinuric nephropathy on dual renin-angiotensin system blockade: the ESPLANADE trial. Clinical journal of the American Society of Nephrology: CJASN. 2010;5(11):1928–38. Epub 2010/07/31. 10.2215/CJN.03380410 20671225 PMC3001777

[25] Ozsoy RC, van der Steeg WA, Kastelein JJ, Arisz L, Koopman MG. Dyslipidaemia as predictor of progressive renal failure and the impact of treatment with atorvastatin. Nephrology, dialysis, transplantation: official publication of the European Dialysis and Transplant Association—European Renal Association. 2007;22(6):1578–86. Epub 2007/03/10. 10.1093/ndt/gfl790 . 17347284

[26] Sandhu S, Wiebe N, Fried LF, Tonelli M. Statins for improving renal outcomes: a meta-analysis. Journal of the American Society of Nephrology: JASN. 2006;17(7):2006–16. Epub 2006/06/10. 10.1681/ASN.2006010012 . 16762986

[27] Bianchi S, Bigazzi R, Caiazza A, Campese VM. A controlled, prospective study of the effects of atorvastatin on proteinuria and progression of kidney disease. American journal of kidney diseases: the official journal of the National Kidney Foundation. 2003;41(3):565–70. Epub 2003/03/04. 10.1053/ajkd.2003.50140 . 12612979

[28] Tonelli M, Moye L, Sacks FM, Kiberd B, Curhan G. Pravastatin for secondary prevention of cardiovascular events in persons with mild chronic renal insufficiency. Annals of internal medicine. 2003;138(2):98–104. Epub 2003/01/17. . 12529091 10.7326/0003-4819-138-2-200301210-00010

[29] Navaneethan SD, Pansini F, Perkovic V, Manno C, Pellegrini F, Johnson DW, et al HMG CoA reductase inhibitors (statins) for people with chronic kidney disease not requiring dialysis. Cochrane database of systematic reviews. 2009;(2):CD007784 Epub 2009/04/17. 10.1002/14651858.CD007784 . 19370693

[30] Rahman M, Baimbridge C, Davis BR, Barzilay J, Basile JN, Henriquez MA, et al Progression of kidney disease in moderately hypercholesterolemic, hypertensive patients randomized to pravastatin versus usual care: a report from the Antihypertensive and Lipid-Lowering Treatment to Prevent Heart Attack Trial (ALLHAT). American journal of kidney diseases: the official journal of the National Kidney Foundation. 2008;52(3):412–24. Epub 2008/08/05. 10.1053/j.ajkd.2008.05.027 18676075 PMC2897819

[31] Shepherd J, Kastelein JJ, Bittner V, Deedwania P, Breazna A, Dobson S, et al Effect of intensive lipid lowering with atorvastatin on renal function in patients with coronary heart disease: the Treating to New Targets (TNT) study. Clinical journal of the American Society of Nephrology: CJASN. 2007;2(6):1131–9. Epub 2007/10/19. 10.2215/CJN.04371206 . 17942759

[32] Campese VM, Park J. HMG-CoA reductase inhibitors and renal function. Clinical journal of the American Society of Nephrology: CJASN. 2007;2(6):1100–3. Epub 2007/10/19. 10.2215/CJN.04060907 . 17942762

[33] Michel O, Heudes D, Lamarre I, Masurier C, Lavau M, Bariety J, et al Reduction of insulin and triglycerides delays glomerulosclerosis in obese Zucker rats. Kidney international. 1997;52(6):1532–42. Epub 1998/01/04. . 9407498 10.1038/ki.1997.483

[34] Rubin R, Silbiger S, Sablay L, Neugarten J. Combined antihypertensive and lipid-lowering therapy in experimental glomerulonephritis. Hypertension. 1994;23(1):92–5. Epub 1994/01/01. . 8282335 10.1161/01.hyp.23.1.92

[35] Diamond JR, Karnovsky MJ. Exacerbation of chronic aminonucleoside nephrosis by dietary cholesterol supplementation. Kidney international. 1987;32(5):671–7. Epub 1987/11/01. . 3430956 10.1038/ki.1987.259

[36] de Zeeuw D, Anzalone DA, Cain VA, Cressman MD, Heerspink HJ, Molitoris BA, et al Renal effects of atorvastatin and rosuvastatin in patients with diabetes who have progressive renal disease (PLANET I): a randomised clinical trial. The lancet Diabetes & endocrinology. 2015;3(3):181–90. Epub 2015/02/11. 10.1016/S2213-8587(14)70246-3 . 25660356

[37] Sarafidis PA, Bakris GL. Does evidence support renin-angiotensin system blockade for slowing nephropathy progression in elderly persons? Annals of internal medicine. 2009;150(10):731–3. Epub 2009/05/20. . 19451583 10.7326/0003-4819-150-10-200905190-00014

[38] Chen JH, Yen YC, Yang HC, Liu SH, Yuan SP, Wu LL, et al Curative-Intent Aggressive Treatment Improves Survival in Elderly Patients With Locally Advanced Head and Neck Squamous Cell Carcinoma and High Comorbidity Index. Medicine (Baltimore). 2016;95(14):e3268 10.1097/MD.0000000000003268 . 27057882 PMC4998798

[39] Chen JH, Yen YC, Liu SH, Yuan SP, Wu LL, Lee FP, et al Outcomes of Induction Chemotherapy for Head and Neck Cancer Patients: A Combined Study of Two National Cohorts in Taiwan. Medicine (Baltimore). 2016;95(7):e2845 Epub 2016/02/18. 10.1097/MD.0000000000002845 . 26886647 PMC4998647

[40] Shao JY, Lee FP, Chang CL, Wu SY. Statin-Based Palliative Therapy for Hepatocellular Carcinoma. Medicine (Baltimore). 2015;94(42):e1801 Epub 2015/10/27. 10.1097/MD.0000000000001801 26496314 PMC4620768

[41] Wu M-S, I-Wen Wu, Hsu K-H. Survival analysis of Taiwan Renal Registry Data System (TWRDS) 2000–2009. Acta Nephrologica 2012;26(2):04–108.

[42] Chiang CE, Wang TD, Ueng KC, Lin TH, Yeh HI, Chen CY, et al 2015 guidelines of the Taiwan Society of Cardiology and the Taiwan Hypertension Society for the management of hypertension. Journal of the Chinese Medical Association: JCMA. 2015;78(1):1–47. Epub 2014/12/31. 10.1016/j.jcma.2014.11.005 . 25547819

[43] Parfrey PS, Foley RN. The clinical epidemiology of cardiac disease in chronic renal failure. Journal of the American Society of Nephrology: JASN. 1999;10(7):1606–15. Epub 1999/07/15. . 10405218 10.1681/ASN.V1071606

[44] Levey AS, Eknoyan G. Cardiovascular disease in chronic renal disease. Nephrology, dialysis, transplantation: official publication of the European Dialysis and Transplant Association—European Renal Association. 1999;14(4):828–33. Epub 1999/05/18. . 10328452 10.1093/ndt/14.4.828

[45] Collins AJ, Foley RN, Herzog C, Chavers BM, Gilbertson D, Ishani A, et al Excerpts from the US Renal Data System 2009 Annual Data Report. American journal of kidney diseases: the official journal of the National Kidney Foundation. 2010;55(1 Suppl 1):S1–420, A6-7. Epub 2010/02/10. 10.1053/j.ajkd.2009.10.009 20082919 PMC2829836

[46] Longenecker JC, Coresh J, Powe NR, Levey AS, Fink NE, Martin A, et al Traditional cardiovascular disease risk factors in dialysis patients compared with the general population: the CHOICE Study. Journal of the American Society of Nephrology: JASN. 2002;13(7):1918–27. Epub 2002/06/29. . 12089389 10.1097/01.asn.0000019641.41496.1e

[47] Landray MJ, Thambyrajah J, McGlynn FJ, Jones HJ, Baigent C, Kendall MJ, et al Epidemiological evaluation of known and suspected cardiovascular risk factors in chronic renal impairment. American journal of kidney diseases: the official journal of the National Kidney Foundation. 2001;38(3):537–46. Epub 2001/09/05. 10.1053/ajkd.2001.26850 . 11532686

[48] Singh S, Singh PP. Statins for prevention of hepatocellular cancer: one step closer? Hepatology. 2014;59(2):724–6. 10.1002/hep.26614 . 23839991

[49] D'Agostino RB Jr. Propensity score methods for bias reduction in the comparison of a treatment to a non-randomized control group. Statistics in medicine. 1998;17(19):2265–81. Epub 1998/11/05. . 9802183 10.1002/(sici)1097-0258(19981015)17:19<2265::aid-sim918>3.0.co;2-b

[50] Schneeweiss S. Sensitivity analysis and external adjustment for unmeasured confounders in epidemiologic database studies of therapeutics. Pharmacoepidemiology and drug safety. 2006;15(5):291–303. Epub 2006/02/01. 10.1002/pds.1200 . 16447304

[51] Yancy CW, Jessup M, Bozkurt B, Butler J, Casey DE Jr., Drazner MH, et al 2013 ACCF/AHA guideline for the management of heart failure: a report of the American College of Cardiology Foundation/American Heart Association Task Force on Practice Guidelines. Journal of the American College of Cardiology. 2013;62(16):e147–239. Epub 2013/06/12. 10.1016/j.jacc.2013.05.019 . 23747642

[52] McMurray JJ, Adamopoulos S, Anker SD, Auricchio A, Bohm M, Dickstein K, et al ESC Guidelines for the diagnosis and treatment of acute and chronic heart failure 2012: The Task Force for the Diagnosis and Treatment of Acute and Chronic Heart Failure 2012 of the European Society of Cardiology. Developed in collaboration with the Heart Failure Association (HFA) of the ESC. European heart journal. 2012;33(14):1787–847. Epub 2012/05/23. 10.1093/eurheartj/ehs104 . 22611136

[53] Jayatilake N, Mendis S, Maheepala P, Mehta FR. Chronic kidney disease of uncertain aetiology: prevalence and causative factors in a developing country. BMC nephrology. 2013;14:180 Epub 2013/08/29. 10.1186/1471-2369-14-180 23981540 PMC3765913

[54] Weiner DE, Sarnak MJ. Managing dyslipidemia in chronic kidney disease. Journal of general internal medicine. 2004;19(10):1045–52. Epub 2004/10/16. 10.1111/j.1525-1497.2004.40049.x 15482558 PMC1492581

[55] Grone EF, Grone HJ. Does hyperlipidemia injure the kidney? Nature clinical practice Nephrology. 2008;4(8):424–5. Epub 2008/07/03. 10.1038/ncpneph0863 . 18594500

[56] Keane WF. Lipids and the kidney. Kidney international. 1994;46(3):910–20. Epub 1994/09/01. . 7996813 10.1038/ki.1994.349

[57] Kim SY, Guijarro C, O'Donnell MP, Kasiske BL, Kim Y, Keane WF. Human mesangial cell production of monocyte chemoattractant protein-1: modulation by lovastatin. Kidney international. 1995;48(2):363–71. Epub 1995/08/01. . 7564103 10.1038/ki.1995.304

[58] Fried LF. Effects of HMG-CoA reductase inhibitors (statins) on progression of kidney disease. Kidney international. 2008;74(5):571–6. Epub 2008/06/06. 10.1038/ki.2008.231 . 18528321

[59] Levey AS, Atkins R, Coresh J, Cohen EP, Collins AJ, Eckardt KU, et al Chronic kidney disease as a global public health problem: approaches and initiatives—a position statement from Kidney Disease Improving Global Outcomes. Kidney international. 2007;72(3):247–59. Epub 2007/06/15. 10.1038/sj.ki.5002343 . 17568785

[60] Cordell HJ. Bias toward the null hypothesis in model-free linkage analysis is highly dependent on the test statistic used. American journal of human genetics. 2004;74(6):1294–302. Epub 2004/05/05. 10.1086/421476 15124101 PMC1182095

