# Supplementary material for: Statins and Renin Angiotensin System Inhibitors Dose-Dependently Protect Hypertensive Patients against Dialysis Risk
Source: PLoS One. 2016 Sep 15;11(9):e0162588. doi: 10.1371/journal.pone.0162588 (PMC5025231; doi:10.1371/journal.pone.0162588)
Supplement: S1 Table — (DOCX) [file pone.0162588.s001.docx]

S1 Table . Candidate Variables for the Logistic Regression Model.

| Candidate variable |  | Odds ratio | 95% Confidence interval | |
| --- | --- | --- | --- | --- |
| Age ≥ 65 years |  | 1.283 | 1.206 | 1.367 |
| Age = 45–64 years |  | 0.629 | 0.423 | 1.191 |
| Female sex |  | 0.736 | 0.710 | 0.821 |
| Diabetes |  | 1.433 | 1.412 | 1.561 |
| Cardiovascular disease |  | 1.679 | 1.612 | 1.778 |
| Cerebrovascular disease |  | 1.320 | 1.171 | 1.452 |
| Liver cirrhosis |  | 1.115 | 1.016 | 1.301 |
| Hepatitis B and C virus infection |  | 1.153 | 1.007 | 1.234 |
| Moderate and severe liver disease |  | 1.042 | 0.861 | 1.323 |
| Asthma |  | 0.987 | 0.876 | 1.125 |
| Dyslipidemia |  | 1.191 | 0.992 | 1.243 |
| Liver cirrhosis |  | 1.223 | 1.162 | 1.294 |
| Urban |  | 0.972 | 0.934 | 1.144 |
| Suburban |  | 0.967 | 0.927 | 1.074 |
| Income ≥ NT$33,301 |  | 0.818 | 0.743 | 0.910 |
| Income = NT$21,000–NT$33,300 |  | 0.946 | 0.857 | 1.049 |
| Income = NT$1–NT$21,000 |  | 0.920 | 0.830 | 1.029 |
| Diuretic use |  | 0.741 | 0.723 | 0.767 |
| Calcium channel blocker use |  | 0.804 | 0.717 | 0.913 |
| Beta blocker use |  | 0.812 | 0.724 | 0.933 |
| Nonstatin lipid-lowering drug use |  | 0.998 | 0.874 | 1.031 |
| Metformin use |  | 1.166 | 1.073 | 1.362 |
| Aspirin use |  | 0.989 | 0.956 | 0.999 |
